# Relationship between Glucose-6-Phosphate Dehydrogenase Deficiency, X-Chromosome Inactivation and Inflammatory Markers

**DOI:** 10.3390/antiox12020334

**Published:** 2023-01-31

**Authors:** Alessandra Errigo, Angela Bitti, Franca Galistu, Roberta Salis, Giovanni Mario Pes, Maria Pina Dore

**Affiliations:** 1 Department of Biomedical Sciences, University of Sassari, 07100 Sassari, Italy; 2 Laboratory of Azienda Ospedaliero-Universitaria, 07100 Sassari, Italy; 3 Dipartimento di Medicina, Chirurgia e Farmacia, University of Sassari, Clinica Medica, Viale San Pietro 8, 07100 Sassari, Italy; 4 Sardinia Longevity Blue Zone Observatory, P.zza Principessa di Navarra, Santa Maria Navarrese, 08040 Baunei, Italy; 5 Department of Medicine, Baylor College of Medicine, One Baylor Plaza Blvd, Houston, TX 77030, USA

**Keywords:** glucose-6-phosphate dehydrogenase, X-chromosome inactivation, inflammatory markers

## Abstract

Recent studies suggest that X-linked glucose-6-phosphate dehydrogenase (G6PD) deficiency entails a proinflammatory state that may increase the risk of several disease conditions. However, it is not clear how this relates to the degree of enzyme insufficiency and, in heterozygous females, to skewed inactivation of the X chromosome. This study aimed to (i) investigate the enzyme activity in a cohort of 232 subjects (54.3% females) from Northern Sardinia, Italy, further stratified into three subgroups (G6PD normal, partial deficiency and total deficiency); (ii) measure the levels of some non-specific inflammatory markers, such as erythrocyte sedimentation rate (ESR), C-reactive protein (CRP), and those derived from cell counts, such as neutrophil-to-lymphocyte ratio (NLR), monocyte-to-lymphocyte ratio (MLR) and platelet-to-lymphocyte ratio (PLR), in relation to the underlying molecular defect and X inactivation. G6PD activity was measured in red blood cells according to G6PD/6PGD ratio, and X-chromosome inactivation was assessed by the HUMARA method. Overall, ESR was increased in males with total deficiency compared with normal males (15.0 ± 7.2 vs. 11.9 ± 6.2, *p* = 0.002, Tukey’s test), albeit not in males with partial deficiency. High-sensitivity CRP was slightly increased in males with total deficiency, compared to males with normal G6PD activity (5.96 ± 3.39 vs. 3.95 ± 2.96, *p* = 0.048). In females, neither marker showed significant differences across the subgroups. MLR was significantly and progressively increased from normal to totally deficient subjects with intermediate values in partially deficient subjects (0.18, 0.31 and 0.37, ANOVA *p* = 0.008). The NLR and PLR were not different in the three subgroups. Our findings show that G6PD deficiency may be associated with a proinflammatory profile, especially in elderly females, and worsened by the concomitant asymmetric inactivation of the X chromosome.

## 1. Introduction

Glucose-6-phosphate dehydrogenase (G6PD, EC 1.1.1.49) is one of the most investigated metabolic enzymes. Its deficiency, largely determined by loss-of-function mutations in the coding gene (OMIM 305900), has been associated with hemolytic anemia and neonatal jaundice following stressful stimuli [1]. Since the G6PD gene maps to the X chromosome, males carrying the mutation are hemizygotes and generally express a totally deficient phenotype, while females carrying the mutation can be homozygotes, showing a phenotype similar to that of hemizygous males, or heterozygotes with a partial-deficiency phenotype [2]. However, it is known that in the somatic cells of normal females, random inactivation of one X chromosome in the pair occurs at an early stage of embryogenesis via epigenetic mechanisms [3]. Consequently, the embryo of a female heterozygote for G6PD mutations is a mosaic of cells in which the transcription of the maternal or paternal X chromosome is randomly silenced. In theory, this phenomenon should lead to 50% of normal cells and 50% of deficient cells, generating an overall phenotype of partial deficiency, with 50% of residual catalytic activity. In reality, given the random nature of the inactivation of the female X chromosome, sometimes deviations from the expected 50% activity can occur [4]. Thus, in heterozygous females, the expression of the gene and, in turn, the range of enzymatic activity follows a continuum, from total deficiency up to full activity. Although these mechanisms have been known for about half a century, and molecular techniques to measure X-chromosome inactivation (XCI) are easily accessible [5], recent studies relating the degree of XCI in females heterozygous for G6PD mutations with the level of the enzyme catalytic activity are rare. Furthermore, in populations where the prevalence of G6PD deficiency is particularly high, it is screened at the neonatal level by semi-quantitative enzyme tests, and often the enzyme activity in the tested sample is expressed in dichotomous form (normal/deficient) or, at most, as a semiquantitative trichotomy (normal/partial/total deficiency). However, the accuracy of these tests in heterozygous females with varying degrees of XCI is unknown [6]. A further complication is given by the fact that nearly 15% of the genes that map to the X chromosome in females “escape” inactivation and are expressed to some extent by the inactive X chromosome [7].

In addition to its role in regulating the redox balance of cells [8], G6PD has also been recognized as having a role in inflammatory processes, although the data in the literature are conflicting. Recently, G6PD has been implicated in several inflammatory diseases, such as cardiovascular disease [9], celiac disease [10] and asthma [11]. Because X-chromosome inactivation increases in elderly females [12], it would be of great interest to understand whether skewed inactivation increases or reduces inflammation markers associated with G6PD deficiency. G6PD promotes inflammation via NF-κB signaling in adipocytes and its expression is increased [13,14,15], while its suppression by knockout or using specific inhibitors decreases the inflammatory response. In G6PD-deficient mice, the expression of a number of cytokines has been reported to be decreased, with the exception of TGF-β and IL-10 [16]. In human skin, inflammatory reactions seem to be dependent on an excessive production of reactive oxygen species due to increased G6PD-generated NADPH [17]. In addition, G6PD inhibition has been shown to reduce the formation of 20-HETE, a potent vasoconstrictor, which affects platelet aggregation [18]. On the contrary, other studies suggest that G6PD deficiency is associated with increased release of proinflammatory cytokines, especially IL-8 [19], and a decreased release of the anti-inflammatory cytokine IL-10 [20,21]. Therefore, the activity of G6PD can have both a pro- and anti-inflammatory effect, depending on the specific cell type and its overall role in the organism [15,22]. These opposing effects could account for the global action of G6PD deficiency in animals and humans, explaining, for example, the fact that a moderate overexpression of G6PD is able to increase the median lifespan in animals [23].

The aims of this study were (i) to investigate the G6PD enzyme activity in a cohort of subjects from Northern Sardinia, Italy, stratified in three subgroups (G6PD normal, partial deficiency and total deficiency) and in females with partial or total deficiency, according to the inactivation pattern of the X chromosome, and (ii) to measure the levels of some non-specific inflammatory markers, such as erythrocyte sedimentation rate (ESR), C-reactive protein (CRP), and those derived from cell counts, such as neutrophil-to-lymphocyte ratio (NLR), monocyte-to-lymphocyte ratio (MLR) and platelet-to-lymphocyte ratio (PLR), in relation to the underlying molecular defect and X inactivation in a cohort of G6PD-deficient subjects of both sexes.

## 2. Materials and Methods

### 2.1. Study Population

In total, 232 subjects, 105 males and 127 females, aged 2 to 96 years, were recruited for the analysis. All subjects were from Northern Sardinia, Italy. Study participants were apparently in good health and none of them showed overt signs or symptoms of acute inflammatory conditions. Since, in females, the skewed inactivation of the X chromosome increases with age, nearly 40% of the entire sample included individuals aged ≥70 years.

### 2.2. Clinical Laboratory

Hematological parameters, including red (RBC) and white blood cell (WBC) counts and hemoglobin (Hb) concentration, were analyzed in each patient by using an ADVIA^®^ 2120i Hematology System (Siemens Healthcare Diagnostics s.r.l., Milano, Italy). G6PD activity was tested in the reference lab (analysis laboratory, Civil Hospital, AOU Sassari).

### 2.3. Determination of G6PD Activity

The G6PD activity was determined by measuring the difference between the catalytic activity of the enzyme and that of 6-phosphogluconate dehydrogenase (6PGD) [2,24,25]. This method improves the sensitivity of the biochemical test and identifies, more accurately, the heterozygote females, with a sensitivity of 85.2% and a specificity of 97.4% [26]. The G6PD/6PGD ratio represents a normalized quantitative value, which avoids the interference of individual variations in the erythrocyte content of Hb, the number of RBCs (for example, in microcytemia and iron-deficient anemia) and the number of reticulocytes and leukocytes. Total G6PD deficiency was defined as G6PD/6PGD percent ratio <10% while the normal value was defined as G6PD/6PGD ≥85% and intermediate between 10% and 85% according to the manufacturer’s instruction (Nurex, Sassari, Italy) based on scientific literature [24,27].

### 2.4. Determination Inflammatory Markers

The erythrocyte sedimentation rate (ESR), a non-specific marker of inflammation, was determined with the Westergren method [28]. Human high-sensitivity C-reactive protein (hs-CRP) was assessed by a commercial ELISA kit (Cusabio, Milano, Italy) following manufacturer’s instructions. Composite systemic inflammatory markers were also mathematically calculated from the blood count variables. The neutrophil-to-lymphocyte ratio (NLR) was calculated as the ratio of neutrophil and lymphocyte count [29,30], the monocyte-to-lymphocyte ratio (MLR) as the ratio of monocyte and lymphocyte count [31] and the platelet-to-lymphocyte ratio (PLR) as the ratio of platelet and lymphocyte count [32].

### 2.5. Identification of G6PD Mutations

Peripheral blood collected from participants was centrifuged at 1200× *g* for 20 min and the buffy coat separated. Genomic DNA was extracted from leukocytes via a standard salting-out protocol, including overnight protease K digestion. The DNA was amplified by PCR for mutations in the G6PD gene using primers described previously [10]. Since previous studies have ascertained a limited number of mutations associated with enzyme deficiency in Sardinia [33,34], the screening focused on the most frequent mutation (G6PD Med, 563 C→T, S188F) and, in case of negative results, the Seattle G6PD mutation (844 G→C, D282H) and G6PD Union mutation (1260 C→T, R454C) were checked, according to the literature [35]. Genotyping of the G6PD 563C→T variant was assessed by restriction fragment-length polymorphism (PCR-RFLP) analysis of PCR-amplified fragments. The G6PD 563T allele, introducing a new MboII restriction site, was analyzed by PCR amplification of exon 6 of the G6PD gene using the oligonucleotide primers 5′-AGGAGGTTCTGGCCTCTACT-3′ and 5′-TGAGGCTCCTGAGTACCACC-3′ followed by digestion with MboII [36].

### 2.6. Determination of X-Chromosome Inactivation Pattern

In female participants, the X-chromosome inactivation pattern was determined using the HUMARA method [37] based on the digestion of exon 1 of the androgen receptor (AR) gene (OMIM 313700), with methylation-sensitive restriction enzymes [37]. Briefly, 50 ng DNA was digested using 10 U/µL HpaII or no enzyme in a 20 µL volume. One µL of DNA was amplified by PCR using the following oligoprimers, sense 5′-TCCAGAATCTGTTCCAGAGCGTGC-3′, labeled with 5′-FAM, and antisense 5′-GCTGTGAAGGTTGCTGTTCCTCAT-3′ [36]. The PCR amplification was performed with the following protocol: initial denaturation at 94 °C for 5 min followed by 30 cycles of 94 °C for 45 s, 60 °C for 30 s and 72 °C for 60 s, followed by a final extension step of 10 min at 72 °C and soaking at 4 °C. The inactivated allele can be easily identified as it is methylated and this prevents cleavage by the restriction enzymes. Fragment separation was obtained by capillary electrophoresis using FAM as fluorescent tags on the Applied Biosystems 310 genetic analyzer (Thermofisher Scientific, Waltham, MA, USA) and each allele was quantified by measuring the peak area with the GeneScan software. Only females heterozygous for AR alleles (CAG repeat) were considered informative, as parental alleles have different lengths. Indicating with *A*1*_d_* and *A*2*_d_* the area of the peak of allele 1 and 2 that was digested, respectively, and with *A*1*_u_* and *A*2*_u_* the areas of the corresponding undigested alleles, the following ratio was calculated:

$$\frac{\frac{A1_{d}}{A1_{u}}}{\frac{A1_{d}}{A1_{u}}+\frac{A2_{d}}{A2_{u}}}$$

A random X-inactivation pattern was defined as a ratio of 50% ± 15%, and a skewed pattern when the ratio was outside this range.

### 2.7. Statistical Analysis

In all study participants, mean values and the standard deviation were calculated for each variable. The Shapiro–Wilk test was used to test the normality of the distribution of blood parameters and inflammation markers. The percent of skewed X inactivation was treated as a continuous variable, with the thresholds mentioned above. The association between study variables and X inactivation was assessed using the Pearson correlation coefficient. Statistical analyses were conducted using SPSS 22.0 (Chicago, IL, USA). For all tests, the cut-off of significance was set to 5%.

## 3. Results

### 3.1. Hematological Parameters of Study Participants

Values of hematological parameters of study participants stratified by subgroups are shown in Table 1. Among 232 study participants, 127 (54.7%) were females. Based on the enzymatic activity, estimated by the G6PD/6PGD ratio, subjects were divided into normal G6PD (females 18%), partially deficient (females 94.4%) and totally deficient (4.8%). Blood parameters and white blood cells counts were substantially comparable across the three groups. However, stratifying by sex, hemoglobin and hematocrit was significantly lower and the mean corpuscular volume greater, in totally deficient males. Basophil count was lower in males with total deficiency, but the small number of subjects did not allow for any definitive conclusions.

The analysis of markers of inflammation by ANOVA showed that the ESR was significantly increased in males with total deficiency compared with normal males (15.0 ± 7.2 vs. 11.9 ± 6.2, *p* = 0.002, Tukey’s test) (Table 1). Among males with partial deficiency, the ESR was slightly increased; however, the small number of subjects did not allow for consistent conclusions. The analysis of hs-CRP revealed a slight increase in males with total deficiency, albeit not in the few males with partial deficiency, compared to males with normal G6PD activity (5.96 ± 3.39 vs. 3.95 ± 2.96, *p* = 0.048, Tukey’s test) (Figure S1). In females, neither marker showed significant differences across the subgroups.

The average values of systemic inflammatory markers NLR and PLR were not different in the three subgroups, while MLR was significantly and progressively increased from normal to totally deficient subjects with intermediate values in partially deficient subjects (0.18, 0.31, and 0.37, ANOVA *p* = 0.008). The MLR correlated positively with age (r = 0.293; *p* < 0.001) and negatively with the residual enzyme activity estimated by the G6PD/6PGD ratio (r = −0.209; *p* = 0.008).

### 3.2. Molecular Analysis

**Figure 1:**
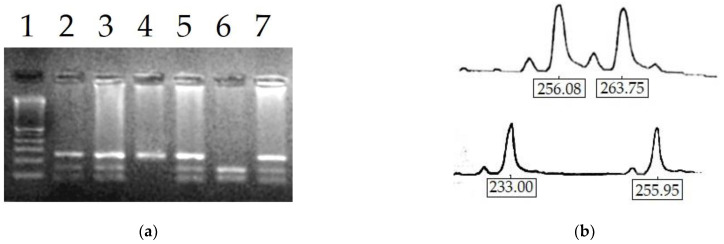
(**a**) Screening of the G6PD Med mutation (563 C→T) by PCR-RFLP (MboII restriction endonuclease). The mutated allele generates two fragments of 145 and 101 bp. Lane 1, marker, lane 2, 3, 5 and 7 heterozygotes females, lane 4, normal, lane 6, hemizygote male (the PCR product band of 246 bp is absent); (**b**) separation of AR alleles by capillary electrophoresis.

Results of the genomic DNA analysis for G6PD mutations are shown in Table 2. The wild-type G6PD B allele was detected in all normal G6PD males; one female was heterozygote for the G6PD Med variant (nucleotide 563 C→T, amino acid S188F) and four females with normal G6PD activity were homozygotes for the wild allele B (Figure 1a). Among the seven hemizygous males with partial deficiency, six showed the common G6PD Med mutation, and one the rarer G6PD Union mutation (nucleotide 1260 C→T, aminoacid R454C). Of the 118 females with partial deficiency, 85 were heterozygotes for the G6PD Med variant, while 9 showed the same variant in homozygosity and 10 were homozygotes for the wild B allele. Furthermore, two females were heterozygous for the G6PD Union mutation, and two were homozygous for the G6PD Seattle variant (nucleotide 844 G→C, amino acid D282H). Among patients with total deficiency, none showed the wild B allele and, as expected, 74 males out of 80 were hemizygotes for the G6PD Med variant, while 6 bore an unknown allele. The four females with a total deficiency were: three homozygous for the G6PD Med variant and one heterozygous for the same variant.

### 3.3. Relationships between XCI Skewing, G6PD Activity and Systemic Inflammation Markers

**Figure 2:**
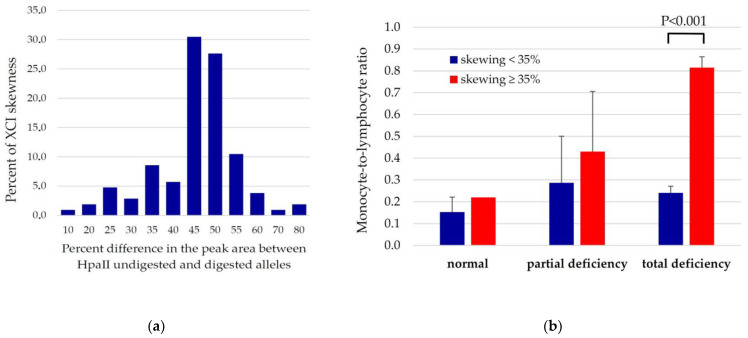
(**a**) Distribution of XCI index among 98 females with G6PD deficiency; (**b**) mean values of monocyte-to-lymphocyte ratio based on G6PD status and percent of X-chromosome skewing. The value of normal females with skewness >35% was estimated from a single subject.

The distribution of ratios between the two AR alleles (Figure 1b) among heterozygous and homozygous females was approximately normal, as shown in Figure 2a. Among 127 females genotyped for HUMARA, only 98 were found to be informative as the remaining were homozygotes for the AR alleles. As expected, a modal value was recorded corresponding to the ratios of 45% and 50%, whereas values lower than 40% or higher than 55% constituted less than 40% of analyzed samples. Pearson’s correlation coefficient between percent of skewness and the age of participants was r = 0.308, *p* = 0.001, attesting to an increased degree of inactivation of the X chromosome in older females.

The degree of XCI differed between the three groups identified by the level of enzyme activity. The systemic inflammatory marker MLR was more than three-times higher in females with total deficiency and skewness ≥ 35% than in normal females (Figure 2b). The regression analysis confirmed a negative association between the G6PD/6PGD ratio and the inflammatory index MLR (standardized β coefficient, −0.171, *p* = 0.043). This association even increased after adjustment for skewness and age (standardized β coefficient, −0.354, *p* = 0.008) (Figure 2b). On the contrary, for NRL, the only significant predictor was age, while for the PRL, any of the two variables were significantly associated.

## 4. Discussion

The G6PD is considered a “housekeeping” enzyme of the pentose phosphate pathway, which generates the NADPH and ribose necessary to synthesize DNA [2]. Loss-of-function mutations in the G6PD gene lead to glutathione depletion and impaired antioxidant defense. Recently, the analysis of large cohorts in a population characterized by a high frequency of G6PD deficiency revealed that this inherited defect, expressed in several tissues in addition to RBCs, was associated with an increased risk of various inflammatory diseases, including cardiovascular [9,10,11]. The clinical severity of G6PD defect is essentially dependent on the type of mutation and the sex-linked transmission. In fact, the defect manifests itself fully in hemizygous males while heterozygous females generally have a less severe phenotype. In a number of sex-related disorders, the preferential skewed inactivation of the mutated allele seems to favor females [38], while in others, the opposite occurs [39]. Regarding the skewed inactivation of the G6PD locus, available data in the literature date back many decades and did not take into account the recent reappraisal of G6PD deficiency as an inflammatory trigger [40]. Lately, studies on the asymmetric inactivation of the G6PD locus in the chromosome pair have been intensified by using more effective methods, such as flow cytometry [41]. In the present study, among 232 patients with G6PD deficiency, only 39.2% were found to have total deficiency while 60.8% had partial deficiency, the latter with a stronger female prevalence. Overall, hematological parameters were similar in normal and deficient subjects; the main difference was a significantly lower number of RBCs among deficient males, as well as a lower concentration of hemoglobin and hematocrit, whereas increased mean corpuscular volume was observed. In our study, two inflammatory markers widely used in clinical practice, i.e., ESR and hs-CRP, were significantly increased in males with total G6PD deficiency compared with normal subjects, while a similar trend was observed in females but without statistical significance. Blood cell counts were used to calculate some systemic inflammation markers that have recently gained importance in clinical pathology [29]. More specifically, the MLR, a combined inflammatory marker that has been extensively used as a predictive marker in a number of systemic inflammatory conditions, being inexpensive and easily derived from blood cell counts, was found to be increased in totally deficient G6PD subjects compared with partially deficient and non-deficient subjects. These findings further support the concept that the enzymatic defect may be associated with an enhanced pro-inflammatory profile in the blood that is consistent with the increased risk of a number of diseases [40]. For example, the MLR has been found increased in rheumatoid arthritis [42], tuberculosis [43], acute gout attack [44] and in several cancers [45,46,47]. In our study, stratifying for age eliminated the association between enzyme level (G6PD/6PGD ratio) and MLR, while adjusting for XCI skewness increased it. This could be explained by assuming that skewed XCI acts as a modifier on the pro-inflammatory effect of G6PD deficiency associated with age. As a consequence, the marked skewness found in elderly females could enhance the proinflammatory impact of G6PD deficiency more than at an earlier age. On the other hand, MLR and PLR did not differ significantly between the subgroups based on the level of catalytic activity. The slightly increased proinflammatory markers in G6PD-deficient subjects found in our study disagree with some literature data that indicate a reduced inflammatory response upon G6PD downregulation [48]. However, in different experimental settings [20,21,49], it has been reported that ex vivo monocytes from subjects with inherited G6PD defects are partially inefficient to inhibit the inflammatory cascade by releasing fewer anti-inflammatory cytokines. In particular, the role of increased TGF-β release by G6PD-deficient cells has been consistently reported [50,51]. These data emphasize the dual effect of G6PD depending on the experimental model and the tissue involved [52].

Regarding the inactivation of the X chromosome in females, our study confirmed the existence of a significant positive correlation between age and skewed inactivation [12,53]. Interestingly, we found a female presenting the normal phenotype, although she was heterozygote for the G6PD Med mutation, suggesting an extremely skewed inactivation of the mutated allele. More difficult to explain is the inactivation pattern in females with a partial deficiency. For instance, despite 64% being heterozygotes for the variant G6PD Med, consistent with the partial phenotype, the remaining 25% did not carry any of the variants screened, suggesting the presence of unknown or missed variants or, less likely, an acquired form of G6PD deficiency [54]. Additionally, 10 females with partial deficiency were genotypically homozygous for the wild-type allele B; these were older females (≥80 years), with one exception, in whom the inactivation of the X chromosome was highly skewed.

G6PD deficiency has been reported in association with increased cardiovascular disease and there are studies in vitro indicating that G6PD deficiency may have an anti-carcinogenetic effect, and in the literature, reports of a risk reduction in patients deficient for G6PD to develop cancer can be found [55,56].

Our study has some limitations. The X-chromosome skewness was determined in peripheral blood leukocytes and may not accurately reflect its extent in other tissues. However, Bittel et al. demonstrated that, despite greater inter-tissue variability, the concordance of XCI between accessible (blood) and inaccessible tissues is high [57]. Moreover, the possibility remains that some study participants were carriers of unknown variants, a situation that can only be confirmed through the systematic sequencing of the whole G6PD gene. As far as inflammation markers are concerned, it cannot be excluded that they may have been influenced by unknown underlying age-related comorbidities, although subjects with acute states or major metabolic diseases, such as diabetes, were excluded during recruitment.

Nonetheless, our study is among the few addressing the consequences of skewing of X chromosome on G6PD activity levels, and is the only one, to the best of our knowledge, that considered the effect of X-chromosome skewness on the pro-inflammatory effect of G6PD deficiency, opening new windows for future research.

In conclusion, the findings of the present study suggest that X-chromosome skewing, associated with normal and deficient G6PD, may partly modulate the pro-inflammatory effect of G6PD deficiency.

## Figures and Tables

**Table 1:** Blood parameters among study participants according to G6PD status.

Parameter	Normal		Partial Deficiency		Total Deficiency	
	Male	Female	Male	Female	Male	Female
No. of cases	18	5	7	118	80	4
Age (mean ± SD, years)	66.8 ± 13.3	59.6 ± 10.9	66.1 ± 13.6	61.9 ± 20.1	58.8 ± 24.7	69.7 ± 12.8
White blood cells (×10^9^/L)	7.33 ± 2.04	5.67 ± 2.24	8.33 ± 3.81	7.68 ± 3.77	8.18 ± 3.22	5.72 ± 2.10
Red blood cells (×10^12^/L)	5.34 ± 0.81	4.63 ± 0.58	4.83 ± 1.02	4.39 ± 0.78	4.41 ± 0.83	4.51 ± 0.46
Hemoglobin (g/L)	14.8 ± 1.5	13.6 ± 1.2	14.2 ± 1.2	12.3 ± 1.7	12.8 ± 2.3 **	13.2 ± 0.8
Hematocrit (%)	43.7 ± 4.4	39.5 ± 3.3	41.9 ± 3.5	37.6 ± 4.8	39.5 ± 6.6 *	40.1 ± 1.7
Mean corpuscular volume (fL)	83.1 ± 10.6	85.6 ± 3.6	88.9 ± 12.0	86.2 ± 11.7	90.4 ± 8.8 *	89.2 ± 5.4
Mean corpuscular hemoglobin (pg)	28.2 ± 4.0	29.4 ± 1.4	30.1 ± 4.0	28.4 ± 4.2	29.4 ± 3.7	29.5 ± 1.2
Mean corpuscular Hb conc. (g/L)	33.8 ± 0.8	34.4 ± 1.8	33.9 ± 1.8	32.6 ± 1.4	32.5 ± 1.4 *	33.1 ± 2.0
Red cell distribution width (%)	14.4 ± 1.9	13.4 ± 2.1	12.9 ± 0.9	13.6 ± 1.8	13.4 ± 2.4	12.5 ± 1.9
Hemoglobin distribution width (g/L)	−	2.67 ± 0.38	3.05 ± 0.92	2.64 ± 0.38	2.66 ± 0.41	2.70 ± 0.6
Platelets (×10^9^/L)	236 ± 42	256 ± 70	220 ± 39	235 ± 75	233 ± 80	170 ± 46
Mean platelet volume (fL)	10.7 ± 1.4	9.84 ± 2.67	10.1 ± 1.4	9.04 ± 1.73	8.5 ± 1.3	11.15 ± 1.64
*White cells count*						
Neutrophils (×10^9^/L)	4.30 ± 1.37	3.21 ± 0.69	5.13 ± 3.61	5.16 ± 3.66	5.24 ± 2.85	3.08 ± 0.31
Lymphocytes (×10^9^/L)	2.33 ± 0.84	1.90 ± 0.77	2.28 ± 0.65	1.76 ± 0.72	2.02 ± 1.04	1.83 ± 0.09
Monocytes (×10^9^/L)	0.42 ± 0.15	0.26 ± 0.05	0.60 ± 0.44	0.45 ± 0.21	0.57 ± 0.27	0.44 ± 0.08
Eosinophils (×10^9^/L)	0.20 ± 0.17	0.19 ± 0.17	0.19 ± 0.21	0.13 ± 0.09	0.17 ± 0.17	0.18 ± 0.17
Basophils (×10^9^/L)	0.07 ± 0.03	0.04 ± 0.04	0.09 ± 0.03	0.04 ± 0.05	0.04 ± 0.05 *	0.07 ± 0.04
Large unstained cells (×10^9^/L)	−	0.11 ± 0.03	0.10 ± 0.01	0.12 ± 0.05	0.14 ± 0.09	0.10 ± 0.02
G6PD (U/gHb)	7.2 ± 2.1	7.4 ± 0.9	4.8 ± 4.6	5.9 ± 2.2	0.6 ± 0.2	0.5 ± 0.8
6PGD (U/gHb)	6.6 ± 0.8	8.0 ± 1.2	7.9 ± 3.2	6.8 ± 2.3	8.8 ± 3.0	7.2 ± 0.5
G6PD/6PGD ratio	0.877 ± 0.033	0.855 ± 0.005	0.220 ± 0.056	0.521 ± 0.187	0.020 ± 0.006	0.115 ± 0.110
*Inflammation markers*						
Erythrocyte sedimentation rate (ESR) (mm/h)	11.9 ± 6.2	13.7 ± 8.9	14.6 ± 9.2	16.1 ± 6.3	15.0 ± 7.2 *	16.0 ± 6.6
High-sensitivity C-reactive protein (hs-CRP) (mg/dL)	3.95 ± 2.96	4.90 ± 3.78	4.40 ± 2.93	5.02 ± 3.46	5.96 ± 3.39 *	7.1 ± 3.2
Neutrophil-to-lymphocyte ratio (NLR)	2.00 ± 0.81	1.87 ± 0.76	2.71 ± 2.93	4.01 ± 5.20	3.47 ± 3.19	1.68 ± 0.08
Monocyte-to-lymphocyte ratio (MLR)	0.18 ± 0.07	0.15 ± 0.07	0.31 ± 0.34	0.31 ± 0.23	0.37 ± 0.26 **	0.34 ± 0.03
Platelet-to-lymphocyte ratio (PLR)	112.8 ± 40.8	153.7 ± 73.2	103.1 ± 30.3	155.7 ± 84.9	141.0 ± 77.4	93.7 ± 30.5

* *p* < 0.05; ** *p* < 0.01 (ANOVA).

**Table 2:** G6PD mutations among study participants according to G6PD status.

Parameter	Normal		Partial Deficiency		Total Deficiency	
	Male	Female	Male	Female	Male	Female
No. of cases	18	5	7	118	80	4
B *	18	−	0	−	−	−
B/B	−	4	−	10	−	0
Med ^#^	0	−	6	−	74	−
B/Med	−	1	−	85	−	1
Med/Med	−	0	−	9	−	3
Union ^§^	0	−	1	−	0	−
B/Union	−	0	−	2	−	0
Seattle/Seattle ^¶^	−	0	−	2	−	0
Unknown	−	−	−	10	6	−

* wild-type G6PD B, ^#^ G6PD Med, 563 C→T, S188F; ^§^ G6PD Union, 1260 C→T, R454C; ^¶^ Seattle G6PD, 844 G→C, D282H.

## Data Availability

Data are contained within the article; however, the data presented in this study will be available on request from the corresponding author.

## Supplementary Materials

The following supporting information can be downloaded at: https://www.mdpi.com/article/10.3390/antiox12020334/s1, Figure S1: Univariate distribution of ESR and serum hs-CRP according to G6PD status.
