# Supplementary figures and images for: Relationship between Glucose-6-Phosphate Dehydrogenase Deficiency, X-Chromosome Inactivation and Inflammatory Markers
Source: Antioxidants (Basel). 2023 Jan 31;12(2):334. doi: 10.3390/antiox12020334 (PMC9952105; doi:10.3390/antiox12020334)

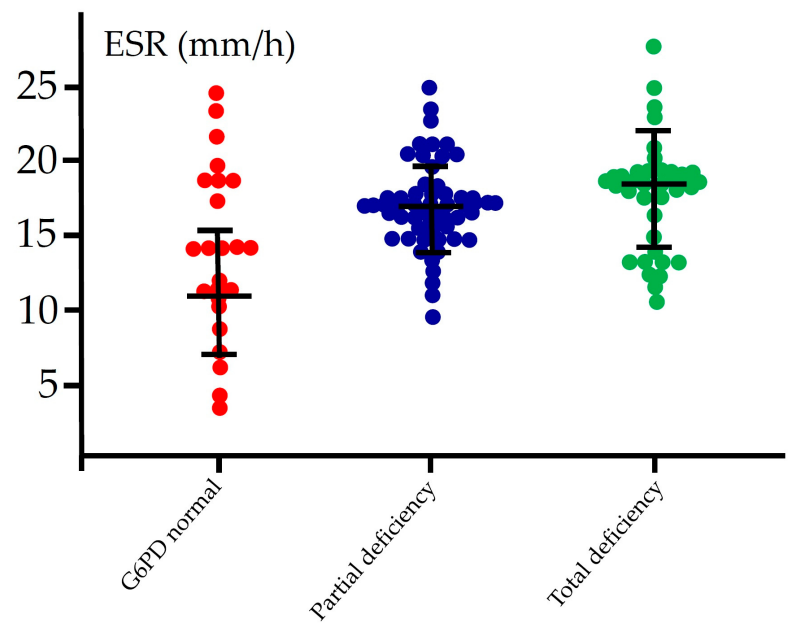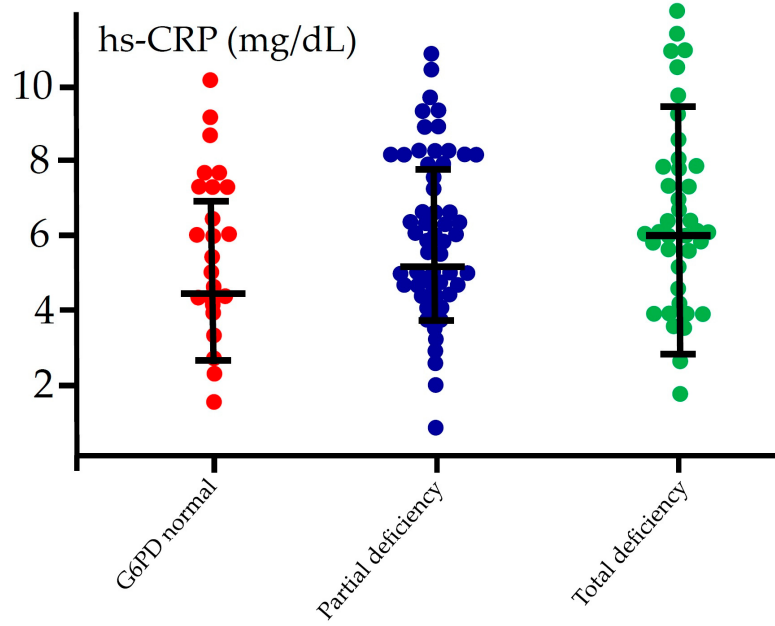

**Figure S1.** Univariate distribution of ESR and serum hs-CRP according to G6PD status.

Supplement: Supplementary file 1 [file antioxidants-12-00334-s001.zip › antioxidants-2134398-supplementary.pdf]
